# Researchers’ Perspectives on Digital Mental Health Intervention Co-Design With Marginalized Community Stakeholder Youth and Families

**DOI:** 10.3389/fpsyt.2022.867460

**Published:** 2022-04-22

**Authors:** Michelle V. Porche, Johanna B. Folk, Marina Tolou-Shams, Lisa R. Fortuna

**Affiliations:** Department of Psychiatry and Behavioral Sciences, University of California, San Francisco, San Francisco, CA, United States

**Keywords:** digital co-design, youth, families, participatory research, marginalized communities, mental health

## Abstract

Co-design of digital mental health technology with youth and families is a relatively new but growing approach to intervention development. In this perspective article, researchers used collaborative reflexivity through duoethnography methodology to reflect and report on experiences and lessons learned conducting co-designed projects with marginalized youth and families. Researchers engaged in written reflective dialogue regarding projects designed to co-develop technology-based apps and computer programs to support mental health of youth and their families. Reflections described the barriers and challenges for sharing responsibilities with stakeholders who have extensive lived experience but limited exposure to research. Researchers shared insights about their own intersectionality and positionality from marginalized to privileged, relative to co-design participants, and what it means to share authority, authentic partnership, and responsibility in the research process. Cultural understanding may diverge, even between acculturated minority researchers and matched minority stakeholders. While there are a variety of approaches that researchers might refer to as co-design, it is important to be intentional in the implementation of these processes so that collaborations with stakeholder youth and families are neither disingenuous nor exploitative. Implications for equitable and meaningful engagement of marginalized communities in co-design projects for youth mental health are discussed.

## Introduction

Co-design of digital mental health technology with youth and families is a relatively new but growing approach to intervention development (1). Marginalized youth and families are often at a disadvantage regarding access to mental health services, due to geographic, cultural, and economic reasons that reflect, and are exacerbated by, systemic racism. These include minoritized (2). Black, Latinx, and Native American individuals, but also those pushed to the margins by socioeconomic and other inequities that limit roles in decision making and creating resources for one’s community. National data on children ages 3–17 collected prior to the ongoing pandemic highlighted rising prevalence rates for anxiety (7.1%) and depression (3.2%), with reduced odds that non-Hispanic Black and lower-income children would receive mental health services (3). Research conducted in the first year of the pandemic revealed increased anxiety and depression for essential frontline workers and their children who were predominantly of Black and Latinx background (4). The ensuing transition to telehealth visits and other digital mental health approaches underscores the urgency of developing effective platforms for service delivery for youth and families.

## Approaches to Co-Design

Co-design involves the inclusion of potential users and stakeholders across the technology development process to ensure the end-product is feasible, acceptable, and effective (5). This approach is a shift from the “expert” or professionally driven design of interventions “for” the users to designing “with” users collaboratively (6). Co-design of interventions to advance health equity has the potential to reduce harm through inclusion and power sharing with people from marginalized communities, who have often been excluded from such processes. It draws on local knowledge and expertise, making space for marginalized voices, to develop more culturally relevant, trusted solutions (7).

A systematic overview of global studies (8) identified that clinical therapy interventions using computer-based cognitive behavioral therapy (CBT) for depression, anxiety, and stress to be as effective as face-to-face CBT, while approaches that use apps and other digital tools for treating the same conditions had mixed results. These highly scalable treatment tools for adolescent anxiety and depression offer avenues for increased access to evidence-based care. A systematic review of recent literature and clinical trials registries worldwide documented 11 mental health interventions planned for youth and families in response to COVID-19, including five designed to deliver CBT or other therapy or support through online computer or app-based platforms (9). No studies reported intervention development using co-design. Co-design processes are often used to promote engagement in digitally delivered interventions with end users, with varying levels of success. Another systematic review of studies examining digital interventions aimed at youth mental health (10) documented six modalities including websites, games and computer-assisted programs, apps, robots and digital devices, virtual reality, and mobile text messaging. This review highlighted a preference by youth for interactive features such as videos, limited text, ability to connect with others, and options to receive text message reminders. The ability to personalize features was also cited as highly desirable (10).

Limited research has been conducted on mental health digital intervention development and implementation with marginalized youth and families. Inequities and the resulting disparities that exist in mental health for a variety of socially marginalized groups have roots in persistent systemic racial bias and underrepresentation in medical and mental health research (11). Inclusion of historically excluded communities is vital to ensuring treatments, including digital interventions, improve access to mental health. The integration of community partnered participatory research, human-centeredness and co-design offers an opportunity for success in this regard (12). Community partnered participatory research stems from the traditions of action research, introduced by Kurt Lewin in the 1940s (13), which blends the social scientific experimental approach with “programs of social action to address social problems” (14). The now broad field of participatory methods includes participatory research, participatory action research, community-based research, action science, action inquiry, and/or cooperative inquiry. These terms are sometimes used interchangeably, however, participatory methods typically represent more emancipatory or transformative action methods, whereas action research is more utilitarian and problem-solving in nature (15). Strong arguments decrying the overwhelming lack of useful clinical research highlights limited patient-centeredness (16). To remedy this deficit, there are various approaches to engage patients in research, of which co-design may be the most ambitious. A review of publications that include the key word “co-design” suggests that the practice has evolved over the last two decades. Early examples include experience-based design to improve patient care through direct personal observation of patient experience (17). By moving beyond consumer reports or satisfaction measures, we gain a deeper understanding of the internal experience, in order to create digital interventions that improve what the individual feels about the tools at a deeper emotional and cognitive level (17, 18).

Participatory approaches to digital health intervention design (19) generally involve stakeholders (e.g., patients, providers, community leaders) in the design process, with the translation of designs to app creation undertaken by individuals with technical expertise (e.g., programmers). These methods are grounded in principles of user-centered design, a well-documented (20, 21) approach that centers the users’ needs by incorporating user-centered activities throughout the development process (22). Allowing end users to influence the design should increase ultimate usability (23). These approaches incorporate various methods to assess intended user needs and preferences, including thorough observation and analysis of tasks and product requirements, development and testing of prototypes, analysis and resolution of usability problems, and iterative testing of features and interfaces (24). These methods are often used to create apps for populations whose users were not involved in the design process, though some approaches involve co-creation of apps for personal use (25).

Participatory informatics is one co-design approach that draws upon principles of Community Partnered Participatory Research (e.g., equity, power sharing) (26) and user-centered design (e.g., active user participation in design) (27), does not require technological expertise, and has been implemented with minoritized populations (28). This approach’s aim is to democratize technology access: end users co-develop the application, including building of the app, through platforms such as Chorus (29), that require no coding expertise to design web-based applications. This method allows non-technical experts to create digital technologies designed to address the gap in availability of appropriate and effective resources that can increase access to benefits from digital health advances. While there are a wide variety of approaches referred to as co-design, researchers must be intentional in the implementation of these processes so that collaboration is not disingenuous. For example, human-centered design approaches privilege the needs of the end users and settings, guarding against over-emphasis on design for clinical trial conditions that ignores realities of complex health care settings (20).

## Methods

Duoethnography, conducted with two or more researchers, as defined by Sawyer and Norris (30) was used to guide this commentary. The authors of this article include a mixed-race Black developmental psychologist, a White clinical psychologist, a clinical psychologist from an immigrant Iranian family, and a bicultural bilingual Latina psychiatrist. As women and caregivers with careers in academic medical research, we have aimed to create opportunities for digital-based intervention development; each of us has experience working with either app-based or computer-based technology for delivery of mental health supports. Interventions include patient navigation and evidence-based treatments (CBT) for youth and families from marginalized communities. For example, the second and third authors’ projects focus on co-designing a mental health services application, exclusively by and for foster care youth, leveraging participatory co-design methods to concurrently expand mental health workforce exposure and capacity by hiring former or current foster care youth as staff within an academic medicine setting. Youth co-designers have increased equity with other staff, faculty, and consultants while receiving unique mental health workforce development opportunities (e.g., resume building, making connections with mental health professionals to promote future career options).

We share a commitment to listening deeply to youth about design of these approaches and in the best of circumstances work to include them in meaningful roles on our research teams. The methodological, practical, and ethical challenges of conducting participatory action research with vulnerable populations, and its value, is well-documented (31). As we gain experience through these projects, we have begun to identify factors that facilitate this work and also areas for improvement, professionally and for the field in general. Because we each have a history of research collaboration that continues to expand, we developed this perspective article using a duoethnography methodology (30) to report and reflect on our experiences and lessons learned while conducting co-designed projects with marginalized youth and families.

After introduction of the inquiry questions and duoethnography process (30), followed by several months of self-reflection and informal conversation, researchers spent 2 months actively engaged in written dialogue, responding to each other regarding projects they are or were involved in (within the previous 5 years) that were designed to co-develop technology to support youth and family mental health. Through turn-taking, researchers responded to previous journal entries in a conversational style, adding new information and engaging in dialogue, yielding the participant data analyzed. Prompts included: What did we learn about working collaboratively with community stakeholders, working with youth and families, marginalized minoritized populations? What does it mean for a researcher to authentically share leadership and design responsibilities with a lay person? How do we address power differentials and the systemic exclusionary context that we are trained in, that is academic research?

Reflexive thematic analysis (32) was conducted with nine entries from the four researchers. A predominantly inductive and experiential orientation was used by the first author. Preliminary codes were developed after multiple readings of the journal entries and then organized into larger themes. Codes and themes as defined and named were then checked for accuracy of meaning in interpretation by the other researchers through examination of written results against codes and raw data. This review was followed by a discussion of the initial summary report and consensus coding. Final results reflect the authors’ collaboratively developed interpretation of identified themes.

## Results

Reflections described the barriers and challenges for researchers sharing responsibilities with co-design partners who have extensive lived experience but limited exposure to research. Reflections also strongly emphasized goals of reaching end users described as racially, ethnically, and linguistically diverse from minoritized communities and families, harmed by systemic racism, silenced, and skeptical about technology use. In describing efforts to respond to apps where “graphics do not usually have individuals or characters that look like my family,” researchers included observations organized into three main themes: (1) *partner characteristics*; (2) *researcher positionality*; and (3) *redefining co-design*.

Co-design partners the researchers had worked with were **characterized** as racially, ethnically and linguistically diverse, from marginalized communities, specifically identifying Latinx youth and families. They varied in interest, skills, and commitment to co-design projects, but overall youth were seen and sought after as sophisticated consumers of technology. A goal of recruitment for co-design partners was representation of the focal end user. One researcher recalled the pain of a youth partner having to grapple with “elements that are offensive” in an effort to culturally adapt an existing mobile treatment program. In acknowledging the challenges for individuals who may be alone, e.g., “the one parent” on a team with researchers, there was a realization of the reluctance partners may feel in asking questions or expressing alternative viewpoints, yet that is essential for collaboration and successful co-development. Cultural considerations were also important, as parent partners may be reluctant to say things that “might be considered disrespectful.” The importance of compensation was noted, with emphasis on hiring partners as project staff. This is aligned with calls for equity in compensation to “community experts” that collaborate with academic researchers (33). There was shared concern and questions about “how do youth define their role?” and we identified the need to be intentional about having these conversations on our projects. The over-reliance on convenience sampling for partners “within reach” suggested the need for improved recruitment strategies.

Recalling previous and current work with co-design partners led to reflections regarding **positionality** as researchers. This included realization of privileged investigator roles where we were more simply “asking for feedback” and confirmation of research questions, with little “sharing of responsibility.” Relying on our own technology expertise hampered the extent to which we tried to obtain “community identification” of both the problems and solutions. A history of researchers “dropping in to take from the community” meant being “met with initial skepticism.” In stepping back, we would want to “check our own views” and more intentionally incorporate cultural humility at the start to examine whether and to what degree partners view technology as the answer.

**FIGURE 1:**
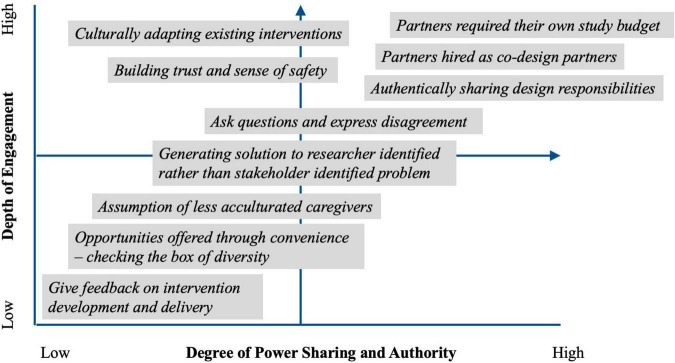
Data from analysis of researcher reflections is used to illustrate a continuum of co-design frameworks that represent (1) the variety of roles for co-design partners and (2) growth as researchers as well as growth in the field toward more authentic and empowered partnerships. These specific examples from the duoethnography analysis are also meant to be used by the reader to reflect and situate their own co-design experiences within this framework.

**Re-defining co-design**, by examining past experiences to inform future aspirations, was a central theme of researcher reflections. Although there was increased clarity regarding the continuum of co-design at different points historically, and for varying goals of co-design for specific projects, a stated goal was for more authentic partnerships that consider both depth of engagement and power sharing by youth and family stakeholders. Codes and quotes derived from the analysis are depicted in Figure 1 along these two continuums to contextualize previous research experiences and future aspirations for co-design. This figure is also meant to encourage the reader to situate and reflect on their own co-design experiences. Fostering authentic partnerships requires “understanding and recognizing power dynamics” that requires “willingness to let go of power and give space” to co-design partners. Researchers described the move toward “community-driven enterprises” where “work together is an exchange between partners.” The boldest vision of this negotiation was with a community-based group that requested resources to investigate their own research questions that would be developed within the co-design process. Although this was a partner-driven request, there was agreement that this is a roadmap for future co-design endeavors. Timing was also a critical aspect of this theme, identifying the need for intentionality in creating guidelines about process and co-design and that these should be discussed as “close to inception” of the research as possible. And finally, there was acknowledgment that co-design, as with other research collaborations, is challenging and time consuming and requires substantial resources to be done well, despite the often-limited research budgets.

## Discussion

Minoritized youth and their families experience multiple barriers to accessing mainstream mental health services, thus collaborative relationships for co-designing services that are accessible, engaging, and respond to patient’s needs and preferences are critical (34). The reflections allowed researchers to explore places in the co-design process where cultural understanding may diverge, even between acculturated researchers from underrepresented backgrounds and matched minoritized stakeholders. Despite a dearth of research on whether co-design improves the clinical effectiveness of interventions, a scoping review of co-design methods with culturally and linguistically diverse communities identified that the quality of the relationship between the researcher and participating community was important for maximizing the community’s experience and engagement with the designed intervention or service (35). These results can facilitate interpretation of the potential of our co-design projects, in advance of end user outcome studies, e.g., the implications for the last author’s implementation of an asynchronous digital evidence-based CBT therapy for childhood anxiety with Latinx and immigrant families. Substantial input from families revealed how child and caregiver would use the intervention together when English proficiency differed between them. Without co-design collaboration the intervention would have been inaccessible, unengaging, difficult to deliver and thus ineffective. Yet, involving families earlier as co-design partners could have facilitated an even more responsive intervention design from the onset.

In our defining of co-design and re-defining our aspirations for its integration in our intervention development, we build on recommendations in the field (36) by highlighting next steps for use that would advance the child and adolescent mental health equity we strive for Harris et al. (37). We suggest a more thorough and intentional practice of transparency in work with youth and families. Incorporating co-design should begin at the earliest possible stage of study design. This may lead to work with various teams in a sequence along the project period, considering youth and family availability, interest, and engagement. Every stage would benefit from explicit naming of roles and expectations for co-design partners, as well as researchers, who should reflect honestly on boundaries regarding power sharing. Part of this self-reflection by researchers should include an understanding of their willingness and ability to embrace the questions and goals of co-design partners. Finally, we encourage a broader strategy for recruitment of partners to increase representation. Networking and relationship building can start even before the proposal writing process and could be a feature of research centers working with youth and families on digital health projects.

We found the duoethnography writing process helpful for sustaining and improving our practice using co-design methods. The act of setting these reflections on paper revealed important themes on positionality and power, and important considerations for fully including youth, family and communities in co-designing technology. The process was an opportunity for increasing self-awareness and learning from colleagues committed to doing this work effectively, justly, and ethically. The central insight that surfaced from the analysis of our data is the enhanced understanding of the dimensions of co-design and what it really means to share the scientific and creative process with youth and families. Diversity and equity practices are a priority and a career-long focus for all of the authors, but frequently research requirements and pressures from academy structures may be a barrier to full participation of youth partners. Examples include limited funding and short timelines that deter potential for meaningful relationship building, as well as bureaucratic tangles that can interfere with incentives and hiring that would more fully promote fairness in co-design partnerships. We learned from this process that we can move beyond these academic norms.

We recommend other researchers engaging in co-design work use similar approaches of collaborative reflexivity. Reflective scientific journaling and dialogue by researchers and partners can evaluate these processes and qualitatively track the dimensions of co-design, positionality, and integration of youth and family prioritized perspectives as end users and scientific partners. More of our scientific reporting can include this information to build the knowledgebase in digital intervention development with marginalized stakeholders, especially if it holds us accountable to striving for optimum co-design that best serves the end user.

## Data Availability Statement

The dataset presented in this article is not readily available because analysis of authors’ reflections of experiences using co-design was conducted using collaborative reflexivity methods. Questions regarding the dataset should be directed to the corresponding author.

## Ethics Statement

Ethical review and approval was not required for the study on human participants in accordance with the local legislation and institutional requirements. Written informed consent from the (patients/participants or patients/participants legal guardian/next of kin) was not required to participate in this study in accordance with the national legislation and the institutional requirements.

## Author Contributions

MP conceptualized and led the duoethnography process and qualitative data analysis. JF contributed to the literature review. All authors participated in reflections and subsequent dialog regarding coding and interpretation, and writing and editing process.

## Conflict of Interest

The authors declare that the research was conducted in the absence of any commercial or financial relationships that could be construed as a potential conflict of interest.

## Publisher’s Note

All claims expressed in this article are solely those of the authors and do not necessarily represent those of their affiliated organizations, or those of the publisher, the editors and the reviewers. Any product that may be evaluated in this article, or claim that may be made by its manufacturer, is not guaranteed or endorsed by the publisher.
